# Associations between physical activity and health-related quality of life among university students in Zhuhai, China

**DOI:** 10.1038/s41598-025-15822-y

**Published:** 2025-08-25

**Authors:** Xiaoyu Tao, Xuelan Wu, Jia Fu, Ying Xiao, Tian Zhong

**Affiliations:** Faculty of Medicine, Macau University of Science and Technology, Avenida WaiLong, Taipa, 999078 Macao S.A.R. People’s Republic of China

**Keywords:** Physical activity, HRQoL, Mental health, University students, Epidemiology, Health care

## Abstract

Physical inactivity is a major global public health concern, contributing to the rising burden of chronic diseases and mental health issues. Despite its known health benefits, physical activity levels remain insufficient, particularly among college students, posing significant risks to their physical and psychological well-being. To address this, a cross-sectional study with 406 university students was conducted in Zhuhai, Guangdong, China, including 280 females and 126 males, all aged between 18 and 21. Physical activity was measured using the International Physical Activity Questionnaire-Short Form (IPAQ-SF), and health-related quality of life (HRQoL) was assessed through the 12-Item Short Form Health Survey (SF-12), which includes Physical Component Summary (PCS) and Mental Component Summary (MCS) scores. Kruskal–Wallis H Tests for differences between groups and Kendall’s Tau-b correlation tests for correlation, revealed that 17.24% of participants had low physical activity levels, with a higher prevalence among female students. Physical activity was positively associated with PCS scores in male students, indicating better physical health with higher activity levels. However, no significant correlation was found between physical activity and MCS scores, suggesting that mental health may be influenced by other factors such as academic stress and social support. These findings emphasize the need for targeted interventions to promote active lifestyles and holistic well-being on campus.

## Introduction

Physical inactivity is a global public health issue with profoundly negative impacts on physical and mental health. Physical inactivity is the fourth leading cause of death globally, as stated by the World Health Organization (WHO)^1^. As growing public concern for health continues to enhance in China, exercise is increasingly being valued. Evidence demonstrates that physical activity enhances physical health and boosts psychological well-being^2^. Social support and policy interventions must be used to encourage physical activity effectively. In 2020, WHO released global physical activity and sedentary behaviour guidelines, referring to physical activity as a “best buy” in public health and urging governments to invest more in associated policies and research to ensure physical activity for everyone^3^. Guidelines on physical activity for patients with chronic conditions were also provided, further reflecting the key position of physical activity in promoting health^4^. In China, increasing physical activity is feasible only through a combination of policy interventions and social support. Research points out that appropriate physical activity reduces the risk of non-communicable diseases significantly and increases overall health levels^5^. Hence, successful policy formulation and implementation, provision of necessary social support, and encouraging more individuals to become physically active are significant for improving public health.

University students’ inactivity physically is strongly related to a number of factors such as mental health, social support, and lifestyle. Physical exercise can lower the anxiety levels of university students indirectly and improve mental health by improving self-efficacy^6^. Physical exercise and health-related quality of life (HRQoL) among university students is an area of much study because of the specific stressors and lifestyle changes encountered by university students. Studies prove that physical activity has a positive impact on HRQoL in university students and that physical activity during leisure time is particularly significant. Studies prove a close connection between physical activity and subjective well-being and life satisfaction among university students. By exercising more, students can enhance life satisfaction and mental health, partly because physical activity promotes psychological resistance and undermines negative affect^7^. Also, exercise can decrease anxiety indirectly and promote mental well-being by increasing self-efficacy and life satisfaction^8^. The intensity and form of exercise also differently affect HRQoL. Findings show moderate - to - vigorous-intensity physical activity is linked with happiness and positive affect among university students, while low-intensity activities such as walking have no significant association^9^. This implies that students must prioritize activities that have the power to contribute significantly to mental and physical health. Finally, leisure-time physical activity is a primary driver for improving HRQoL. It has a robust positive correlation with physical and mental well-being, while work-related physical activity can compromise HRQoL^6^. Therefore, encouraging university students to be engaged in sports during their leisure time can help improve their HRQoL and overall well-being.

## Methods

### Study design and sample

This study was a cross-sectional investigation that aimed to examine the relationship between physical activity (PA) levels and health-related quality of life (HRQOL) among university students in Zhuhai, Guangdong, China. The study participants were enrolled students from universities in Zhuhai, who had been recruited using a stratified random sampling method. In order to ensure representativeness and diversity of the sample students were stratified by academic year and major and random sampling was conducted within each strata. The sample size was calculated assuming an α = 0.05 and an expected low PA prevalence of 50%, obtaining a final sample size of 427 individuals. To be included in the study, participants had to be aged 18–21 years, had normal physical function, and participated voluntarily. Exclusion criteria for the study were clear and definitive: individuals not within the 18–21 age range, those with abnormal physical function or pre-existing conditions impacting participation, and individuals without voluntary consent. Additionally, the total duration of PA per day was more than 16 h, or if the answers contained missing data were excluded.

### Data collection

Data were gathered using an online survey by Questionnaire Star (Changsha Ranxing Information Technology Co., Ltd.). We used this reputable online survey software to create a website for the survey. Sociodemographic characteristics, PA examination, and HRQOL analysis were all included in the survey. Sociodemographic characteristics assessed included age, gender, grade,specialty, and BMI. Specialty were classified into “health related” (clinical medicine, health management, nursing, and health-related specialties ), “non-health related”. All participants had to learn and consent to an educated consent form before answering the questionnaire. All information had been anonymized to protect the participants’ privacy.

### Measurement tools

The International Physical Activity Questionnaire- Short Form (IPAQ- SF)^10^, which evaluated physical activity over the past seven days, particularly vigorous and moderate exercise, jogging, and passive period, was used to determine PA levels. The IPAQ guidelines determined PA levels based on the metabolic equivalent values (MET-min/week). The 12-Item Short Form Health Survey (SF-12), which included two dimensions, physical health (PCS) and mental health (MCS), was used to measure HRQOL. A PCS rating of 50 indicated bad physical health, while a 42 MCS report indicated possible mental health issues. Data processing and analysis were performed according to the IPAQ-SF guidelines^11^.

### Data processing and statistical analysis

There were 440 completed questionnaires, of which 34 had been excluded due to irregularities, giving 406 valid responses (92.27%). Statistical analysis was performed using SPSS 26.0 software (IBM SPSS 26.0, SPSS Inc.), with a significance level set at *P* < 0.05. All data distributions were found to be not-normal according to the Shapiro–Wilk test. Descriptive statistics had been used to summarize continuous variables as median ± interquartile range, shown as M(Q1,Q3), while categorical variables had been presented as frequency and percentage (N, %). Group comparisons had been conducted using the Kruskal–Wallis H test, Mann–Whitney U test, and Pearson’ s chi-square test. Differences in PCS and MCS scores across PA levels had been analyzed using Kruskal–Wallis H Test for differences between groups. The correlation between PA levels and HRQOL had been assessed using Kendall’s Tau-b correlation analysis.

### Ethical approval

This study had been approved by the Research Ethics Committee of the Faculty of Medicine at Macau University of Science and Technology (Approval Number: MUST-FMD-20231114001). All methods were performed in accordance with the relevant guidelines and regulations. All participants had provided written informed consent and had been informed of their right to withdraw from the study at any time. Data had been strictly anonymized to ensure privacy protection. Standardized measurement tools and rigorous statistical analyses had been employed to enhance the scientific validity and reliability of the study.

## Results

### Demographic characteristics

The study included 406 participants, with 280 females and 126 males. The average age was 20.39 years. The details as shown in Table 1.

**Table 1:** Characteristics of participants stratified by gender.

Variable	Total	Male	Female
Specialty, N (%)			
Health related	114(28.08)	21(16.67)	93(33.22)
Non-Health related	292(71.92)	105(83.33)	187(66.79)
Grade, N (%)			
Freshman	159(39.16)	56(44.44)	103(36.79)
Sophomore	136(33.50)	46(36.51)	90(32.14)
Junior	61(15.02)	12(9.52)	49(17.50)
Senior	50(12.31)	12(9.52)	38(13.57)
BMI categories, N (%)			
Underweight	99(24.38)	19(15.08)	80(28.57)
Normal weight	221(54.43)	72(57.14)	149(53.21)
Overweight	34(8.37)	16(12.70)	18(6.43)
Obese	52(12.81)	19(15.08)	33(11.79)
MET-min/week, M(Q1,Q3)^a^	2373.00(1118.40, 4236.50)	3338.95(1484.25, 5715.00)	1962.50(991.00, 3602.00)

^a^Metabolic equivalent minutes per week (MET-min/week), was used to assess the physical activity levels.

The majority of participants had a normal BMI, but 26.11% were classified as overweight or obese. There was a statistically significant difference in BMI categories between genders (*P* < 0.01). Female students had a higher prevalence of being underweight. No significant differences in BMI categories were found based on academic specialty. The details as shown in Table 2.

**Table 2:** Demographics by BMI categories and the analysis results.

Characteristics	BMI Categories^21^					x^2^-value
	Underweight	Normal	Overweight	Obese	$\nu$	
Gender					3	11.55**
Male	19	61	26	20		
Female	80	140	26	34		
Specialty					3	3.77
Health related	22	70	10	12		
Non-health related	77	151	24	40		
Grade					9	15.90
Freshman	41	79	17	22		
Sophomore	32	70	11	23		
Junior	17	34	5	5		
Senior	9	38	1	2		

**P < 0.01.

^a^The values are reported in absolute frequency.

### Participants’ physical activity levels

43.35% of participants engaged in moderate physical activity, while 39.41% engaged in high physical activity. A statistically significant difference (*P* < 0.001) in physical activity levels was observed between genders, with female students having a higher prevalence of low physical activity. The details as shown in Table 3.

**Table 3:** Demographics by physical activity level and the analysis results.

Characteristics	Physical activity level^a^			$\nu$	X^2^-value
	Moderate	High	Low		
Gender				2	21.49***
Male	20	36	70		
Female	50	140	90		
Specialty				2	3.37
Health related	11	58	45		
Non-health related	59	118	115		
Grade				6	15.39
Freshman	33	55	72		
Sophomore	19	59	58		
Junior	9	30	11		
Senior	70	176	160		

***P < 0.01.

^a^The values are reported in absolute frequency.

### Health-related quality of life of participants

The mean Physical Component Summary (PCS-12) score was 50.09 ± 6.31. It was higher in males (51.18 ± 6.98) than in females (49.59 ± 5.93; *P* < 0.01). The mean Mental Component Summary (MCS-12) score was 45.01 ± 8.75. The mean scores for males and females were 45.40 ± 8.70 and 44.83 ± 8.79, respectively, with no significant difference between groups.

### Physical activity and health-related quality of life

No significant correlation was found between physical activity levels and overall PCS-12 or MCS-12 scores. However, among male students, those with low physical activity had significantly lower PCS-12 scores compared to those with moderate and high physical activity levels (*P* < 0.05). The details as shown in Table 4.

**Table 4:** PCS-12 scores of respondents according to different PA groups.

PAL	n	PCS-12^a^	*df*	H
Total			2	2.62
Low	70	50.00(45.81, 54.13)		
Moderate	176	50.64(45.86, 54.29)		
High	160	52.12(46.17, 55.84)		
Male			2	5.87*
Low	20	47.56(41.70, 64.94)		
Moderate	36	52.68(49.44, 56.89)		
High	70	53.57(47.22, 56.84)		
Female			2	0.53
Low	50	50.33(47.94, 54.13)		
Moderate	140	50.01(45.19, 54.19)		
High	90	50.38(45.58, 54.23)		

^a^Data was shown as M(Q1,Q3).

## Discussion

This study found that 17.24% of participants exhibited low physical activity levels, with 15.87% of males and 17.86% of females in this category. Compared to some other studies, the prevalence of low physical activity levels in this study actually appears to be relatively low. For instance, a systematic review and meta-analysis indicated that physical activity was positively associated with quality of life among college students without comorbidities for cardiometabolic diseases, and the levels of physical activity in some studies were also found to be relatively high^12^. The lower physical activity levels among university students compared to the general population may relate to multiple factors. During the transition to higher education, students often develop unhealthy habits such as physical inactivity and poor dietary choices, which may persist for years and elevate risks of non-communicable diseases (NCDs)^13^. Declines in physical activity levels may stem from personal and interpersonal barriers, including academic stress, perceived lack of skills, limited social connections, and peer influences^14^. However, it is worth noting that most students in our study still showed physical activity levels that are more beneficial than harmful to health, which could shift the direction of the discussion.

Institutions may use focused strategies to increase student physical activity rates. It is crucial to improve access to sporting features. According to studies, student participation patterns are considerably influenced by administrative support, resources, and organized opportunities for physical activity^15^. Similarly crucial is successful health education. Universities may encourage more students to engage in physical activity by teaching them about exercise’s cardio and holistic health benefits^16–18^. Furthermore, a study highlighted the effectiveness of interventions targeting physical activity, nutrition, and healthy weight for university and college students, suggesting that such interventions can significantly improve physical activity levels and related health outcomes^15^.

When examining the relationship between physical activity levels and health-related quality of life (HRQoL) among male students, a significant association was observed between physical activity and physical health scores (PCS), suggesting that lower activity levels may contribute to poorer physical health^19^. These results contrast with other studies that show that a decline in physical activity is related to a decline in lifestyle pleasure, happiness, and overall health status^20^. More research indicates that inappropriate physical activity may result in mental health issues like depression and anxiety^21^. A multinational study of university students found that sedentary behaviour was linked to lower living pleasure and enjoyment. Moreover, moderate or vigorous physical activity increases the likelihood of better health, enjoyment, and overall well-being^20^. This implies that encouraging physical activity may promote well-being and improve mental health and overall quality of life.

Another research points out that lower physical activity ranges are related to mental health problems such as stress, anxiety, and depression^22^. A meta-analysis of the effects of physical exercise on anxiety symptoms of college students revealed that exercise interventions can play a certain role in reducing psychological anxiety symptoms, with different exercise modes, durations, and intensities having varying effects^23^. Additionally, according to the study, women typically engage in mild to vigorous exercise more sparingly than men, a gap that several factors may influence. Biological variations may be a factor. For example, female recovery occurs more quickly after exercise, which might impact their choice of intensity^24^. Sex preconceptions may also influence participation designs. This problem is made even more complicated by economic boundaries. In high-intensity activities, women frequently experience heightened political system image anxiety, which may hinder them from engaging in such activities. Women’s opportunities to engage in various physical activities may also be constrained by issues relating to convenience and protection of exercise facilities^25,26^. Furthermore, a study on physical activity and mental health in undergraduate students indicated that high levels of leisure-time physical activity and moderate levels of occupational physical activity are associated with better mental health, highlighting the importance of promoting physical activity in different domains^27^.

## Conclusion

This study highlighted a significant correlation between physical activity levels and HRQoL among Zhuhai University students. The results indicated that while physical activity was positively associated with better physical health benefits, its effected on mental health remain complicated and may be influenced by extra components such as academic stress and social support. Importantly, adult students with lower levels of physical activity showed considerably worse physical health, highlighting the importance of encouraging regular exercise among college students.

Institutions should actively encourage physical activity among students, given the prevalence of sedentary habits and the health risks of physical inactivity. The threats of physical inactivity may be reduced by improving access to sports services, promoting education programs on the benefits of physical activity, and incorporating planned exercise opportunities into educational settings. Also, implementing nutrition policies and promoting healthy eating habits through improved meal options in university cafeterias can help students stay healthy.

To handle the emotional issues that college students face, mental health treatments like peer support and counselling services should also be prioritized. According to the results, a complete approach that includes social and academic aid increased students ' overall quality of life even though physical activity alone may not immediately affect mental health.

Future studies may look at longitudinal changes in school students ' physical activity and HRQoL to better understand direct associations. Moreover, examining the influence of institutional and environmental factors on student physical activity behaviours could aid in creating focused health promotion strategies.

## Supplementary Information

Supplementary Information.

## Data Availability

The data underlying this study are available in the supplementary materials section of this article. All data files are provided in English.
